# Eliminating left ventricular outlet stenosis lowers the risk for endocardial fibroelastosis recurrence^†^

**DOI:** 10.1093/ejcts/ezaf214

**Published:** 2025-06-26

**Authors:** Gregor Gierlinger, Daniel Diaz-Gil, Andreas Tulzer, Roland Mair, Eva Sames-Dolzer, Kerstin Saraci, Steven J Staffa, David Zurakowski, Michaela Kreuzer, Fabian Seeber, Sitaram M Emani, Pedro J del Nido, Rudolf Mair, Ingeborg Friehs

**Affiliations:** Department of Cardiac Surgery, Boston Children’s Hospital, Boston, MA, USA; Division of Pediatric and Congenital Heart Surgery, Kepler University Hospital, Linz, Austria; Medical Faculty, Johannes Kepler University Linz, Linz, Austria; Department of Cardiac Surgery, Boston Children’s Hospital, Boston, MA, USA; Department of Pediatrics, Boston Children’s Hospital, Boston, MA, USA; Department of Pediatrics, Harvard Medical School, Boston, MA, USA; Department of Pediatrics, Boston University Chobanian & Avedisian School of Medicine, Boston, MA, USA; Department of Pediatric Heart Medicine and Adults with Congenital Heart Disease, University Heart and Vascular Center, University Medical Center Hamburg-Eppendorf, Hamburg, Germany; Medical Faculty, Johannes Kepler University Linz, Linz, Austria; Children's Heart Center Linz, Department of Pediatric Cardiology, Kepler University Hospital, Linz, Austria; Division of Pediatric and Congenital Heart Surgery, Kepler University Hospital, Linz, Austria; Division of Pediatric and Congenital Heart Surgery, Kepler University Hospital, Linz, Austria; Medical Faculty, Johannes Kepler University Linz, Linz, Austria; Department of Cardiac Surgery, Boston Children’s Hospital, Boston, MA, USA; Department of Anesthesiology, Critical Care, and Pain Medicine, Boston Children's Hospital, Boston, MA, USA; Department of Anesthesiology, Critical Care, and Pain Medicine, Boston Children's Hospital, Boston, MA, USA; Department of Anesthesia, Harvard Medical School, Boston, MA, USA; Division of Pediatric and Congenital Heart Surgery, Kepler University Hospital, Linz, Austria; Medical Faculty, Johannes Kepler University Linz, Linz, Austria; Division of Pediatric and Congenital Heart Surgery, Kepler University Hospital, Linz, Austria; Department of Cardiac Surgery, Boston Children’s Hospital, Boston, MA, USA; Department of Surgery, Harvard Medical School, Boston, MA, USA; Department of Cardiac Surgery, Boston Children’s Hospital, Boston, MA, USA; Department of Surgery, Harvard Medical School, Boston, MA, USA; Division of Pediatric and Congenital Heart Surgery, Kepler University Hospital, Linz, Austria; Department of Cardiac Surgery, Boston Children’s Hospital, Boston, MA, USA; Department of Surgery, Harvard Medical School, Boston, MA, USA

**Keywords:** Critical aortic stenosis, Endocardial fibroelastosis, Borderline left ventricle, Ross/Ross-Konno, Staged ventricular recruitment, Hypoplastic left heart

## Abstract

**OBJECTIVES:**

Patients with endocardial fibroelastosis (EFE) in the setting of congenital critical aortic valve (AoV) stenosis and left ventricular outflow tract obstruction (LVOTO) are at risk for diastolic dysfunction, limiting biventricular circulation. EFE resection is the only available treatment option, but frequently recurs requiring re-resections. We aimed to investigate whether augmentation of a left ventricular outlet stenosis (AoV stenosis ± LVOTO) with a Ross/Ross-Konno procedure prevents EFE recurrence.

**METHODS:**

Patients born with AoV stenosis ± LVOTO and treated with primary left ventricular (LV) EFE resection at the study centres from January 2010 to December 2021 were included in the study. The inclusion criteria for this retrospective analysis was the presence or absence of a Ross/Ross-Konno procedure for the treatment of a modifiable risk factor of EFE recurrence. Retrospective allocation to either the non-Ross or Ross/Ross-Konno group was carried out accordingly. The primary outcome measure was EFE recurrence.

**RESULTS:**

Ninety-three patients were screened, and 60/93 patients (64.5%) met all inclusion criteria. Within those 60 patients, 5/23 (20.7%) in the Ross/Ross-Konno group had EFE recurrence compared to 23/37 (62.2%) in the non-Ross group [difference = 40.5%, 95% confidence interval (CI) 14.6–58.7, *P *= 0.003] and were less likely to develop EFE recurrence with adjusted hazard ratio of 4.07 (95% CI 1.38–12.0, *P *= 0.011) and 3.69 (95% CI 1.31–10.42, *P *= 0.014) when including death as a competing event.

**CONCLUSIONS:**

This study found that patients after a Ross/Ross-Konno procedure were less likely to experience EFE recurrence and reinterventions on the LVOT/AoV were significantly reduced. However, patient selection and timing of a Ross/Ross-Konno procedure to prevent EFE recurrence have yet to be identified through prospective trials.

**IRB PROTOCOL NUMBERS:**

IRB-P0038762 approved 4/29/2021 (Boston Children’s Hospital) and Ek Nr: 1137/2023 approved 10/25/2023 (Medical Faculty of Johannes Kepler University Linz).

## GRAPHICAL ABSTRACT

**Figure:**
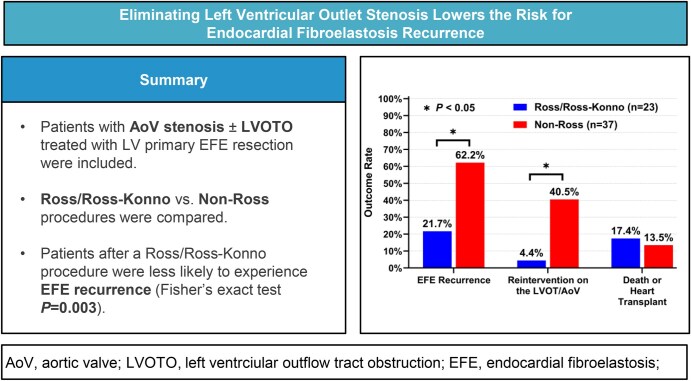

## INTRODUCTION

Congenital critical aortic stenosis (AS) in neonates and young infants presents a serious clinical challenge that necessitates immediate intervention with repeated balloon dilations, aortic valve (AoV) repair or employing the Ross/Ross-Konno procedure. This congenital defect is associated with a significant risk of mortality, primarily due to related complex cardiac anomalies such as a borderline left ventricle (LV), endocardial fibroelastosis (EFE) and mitral valve defects [1–4]. Despite a range of early survival rates reported from 78% to 100% [5–10], the long-term outlook remains concerning, with 10-year survival rates as low as 56% [5–13]. Patients often endure severe LV diastolic dysfunction, which leads to frequent reinterventions and diminished exercise capacity, even after successful alleviation of the aortic obstruction [14–17]. EFE is a well-known marker of adverse outcomes despite successfully relieving the aortic obstruction, as it exerts restrictive forces on the LV myocardium and significantly contributes to diastolic dysfunction [18, 19]. Robinson *et al.* [17] reported that LV diastolic dysfunction was not related to the patient’s age, residual obstruction or left heart dimensions but rather thought to be a consequence of restrictive physiology imposed by EFE on the LV.

EFE develops *in utero* and consists of rigid tissue that inhibits left ventricular (LV) growth [20], contributes to diastolic dysfunction [17] and, thus, impedes the feasibility of postnatal curative biventricular repair. LV EFE resection was, therefore, introduced in neonates and infants suffering from congenital AoV disease and LVOT obstructions to promote the potential for LV growth and preservation of diastolic function. Resection of EFE is currently the only available treatment option and is implemented in early biventricular management, as previously described staged LV recruitment strategy at our centre [9, 21, 22]. However, EFE often recurs after primary resection and infiltrates into the underlying myocardium [23].

Our previous research involved predictive modelling that pinpointed several risk factors for early recurrence of EFE, including structural abnormalities from defective heart valves, chronic pressure overload due to persistent AoV annular restriction from birth, flow disturbances and genetic predisposition [24]. With the success of foetal aortovalvotomy to prevent the progression of EFE in evolving hypoplastic left heart syndrome (HLHS) in mind [25], we first focused on the surgical relief of left ventricular outlet stenosis (AoV stenosis ± LVOTO) [26].

We performed a retrospective analysis comparing a patient cohort that underwent surgical augmentation of the left ventricular outlet stenosis with the Ross/Ross-Konno procedure with an anatomically and haemodynamically matched cohort managed with repair of the AoV and LVOT. The aim of this study was to evaluate whether a Ross/Ross-Konno procedure could prevent EFE recurrence following primary LV EFE resection.

## METHODS

### Study design and patient selection

Patients diagnosed with congenital critical AoV stenosis ± LVOT obstruction at birth accompanied by EFE who were treated with primary EFE resection at Boston Children's Hospital, USA, or Kepler University Hospital, Linz, Austria from January 2010 to December 2021, were included in this study. Exclusion criteria were prior EFE resection in the LV either at the study sites (before 2010) or at an external institution, echocardiographic hypoplastic LV (LVED long axis z-score < −2) or severely depressed LV function prior to the primary EFE resection procedure.

The aim of this study was to determine whether EFE recurrence could be altered if one of the modifiable risk factors identified in our previous publication [24] was successfully addressed. The modifiable risk factor analysed in this manuscript was the gradient across the LV outflow tract and long-term preoperatively reduced AoV valve diameter with long-standing pressure overload as a consequence, which was associated with faster recurrence of EFE. Thus, we retrospectively gathered data from the medical charts of both centres on all patients undergoing EFE resection with or without surgical repair of the AoV diameter through a Ross/Ross-Konno procedure. We did not address why the outflow tract procedure was chosen; instead, we used the presence or absence of a Ross/Ross-Konno procedure as the basis for assignment to either group. This provided us with an opportunity to compare well-balanced, unbiased groups based on anatomical and haemodynamic parameters, allocated according to the presence or absence of a Ross/Ross-Konno procedure, in a retrospective study design. In brief, our centres’ indications for a Ross/Ross-Konno procedure depended on the functionality of the AoV plus the ability to perform a sustained AoV repair or subaortic resection. A detailed description of the surgical technique of the Ross-Konno procedure was recently published by our group [9].

Medical records were reviewed, and relevant data were extracted regarding demographics and previous patients' history. The diagnosis and degree of EFE was reassessed through measurements of preoperative echocardiography and cardiac magnetic resonance (CMR). The extent and location of EFE resection were documented by reviewing surgical notes. Postoperative (predischarge) echocardiographic images were reviewed for degree of residual EFE within the LV, LV function and residual mitral valve disease. The latest follow-up echocardiography images were reviewed for EFE recurrence. If patients had a second EFE resection within the follow-up period, echocardiography, CMR and surgical notes were additionally reviewed at this time point (Supplementary Material, Fig. S1). Additional surgical interventions in the follow-up period were documented, focusing on reoperations on the AoV, LVOT, mitral valve, or single to biventricular conversion surgeries.

Patients were evaluated if a physiologic circulation (right ventricle = sub-pulmonary ventricle and LV = sub-systemic ventricle) could be achieved taking into account adverse outcomes such as death, heart transplantation, left ventricular assist device implantation, LV used as sub-pulmonary ventricle (reversed ventricle repair strategy) or pursuit of definite single ventricle pathway (complete resection of atrial septum). This study did not aim to investigate anatomical or hemodynamical parameters to decide if patients were suitable for a biventricular repair. Furthermore, the decision-making on when to convert to a biventricular circulation was centre-specific. The extension of the Ross procedure to include a modified Konno was at the surgeon's discretion regarding the necessity to enlarge the LVOT.

This joint retrospective study was approved by the Institutional Review Board at Boston Children’s Hospital (Protocol: IRB-P0038762 approved 4/29/2021) and the ethics committee of the Medical Faculty at Johannes Kepler University Linz (EK Nr: 1137/2023 approved 10/25/2023); written patient informed consent was waived.

### EFE diagnosis, grading and independent evaluation of EFE recurrence

A detailed description can be found in the Supplemental Materials.

### Statistical analysis

Data are reported as median (interquartile range, IQR) for continuous variables or frequency (%) for categorical variables. The primary outcome measure was EFE recurrence during the follow-up period and was derived from the independent, blinded evaluation of echocardiography at the last available follow-up or before a second EFE resection surgery compared to the postoperative predischarge echocardiography. Patients who underwent a Ross/Ross**-**Konno procedure at the time of primary EFE resection or at any time point before were compared to patients who had not undergone a Ross/Ross**-**Konno procedure at the time of primary EFE resection. EFE recurrence was compared with Fisher’s exact test and differences in outcome rates were calculated with corresponding 95% confidence intervals (CI).

Multivariable Cox regression analysis of EFE recurrence and multivariable competing risks regression analysis using the Fine-Gray model to account for mortality as a competing event were implemented. A comparison of patients was made for those who received a Ross/Ross**-**Konno procedure vs non-Ross patients in regards to gender, foetal aortic balloon valvuloplasty, neonatal aortic balloon valvuloplasty, AoV annulus z-score, mitral valve area z-score, LV global function before primary EFE resection, LV long-axis z-score, mitral valve disease (including mitral valve surgery at any time in life or residual ≥ moderate mitral valve stenosis or insufficiency after primary EFE resection), age at primary EFE resection, EFE grade [1–3], and LV dysfunction after primary EFE resection. Standardized mean differences (SMDs) were calculated to determine balance between the two groups regarding these potential confounders. A SMD ≥ 0.2 is considered to indicate an imbalance for a given variable, and these variables were included in the multivariable adjustment. Multivariable competing risks analysis and Cox modelling results are reported as adjusted hazard ratios with corresponding 95% CI and *P*-values. Kaplan–Meier curves were constructed to estimate cause-specific freedom from event since primary EFE resection, with group comparisons performed using the log-rank test [27, 28]. For secondary outcomes, patients were compared with respect to their need for reintervention on the LVOT and/or AoV and successful physiologic biventricular management as the ultimate goal.

Statistical analysis was performed using Stata (version 18, StataCorp LLC, College Station, TX). A two-tailed *P *< 0.05 was considered statistically significant. There was no prespecified plan to adjust for multiple comparisons to avoid an increased risk of false negatives and to maintain statistical power [29] when exploring clinically significant differences in EFE recurrence rates between the study groups.

## RESULTS

**Figure 1:**
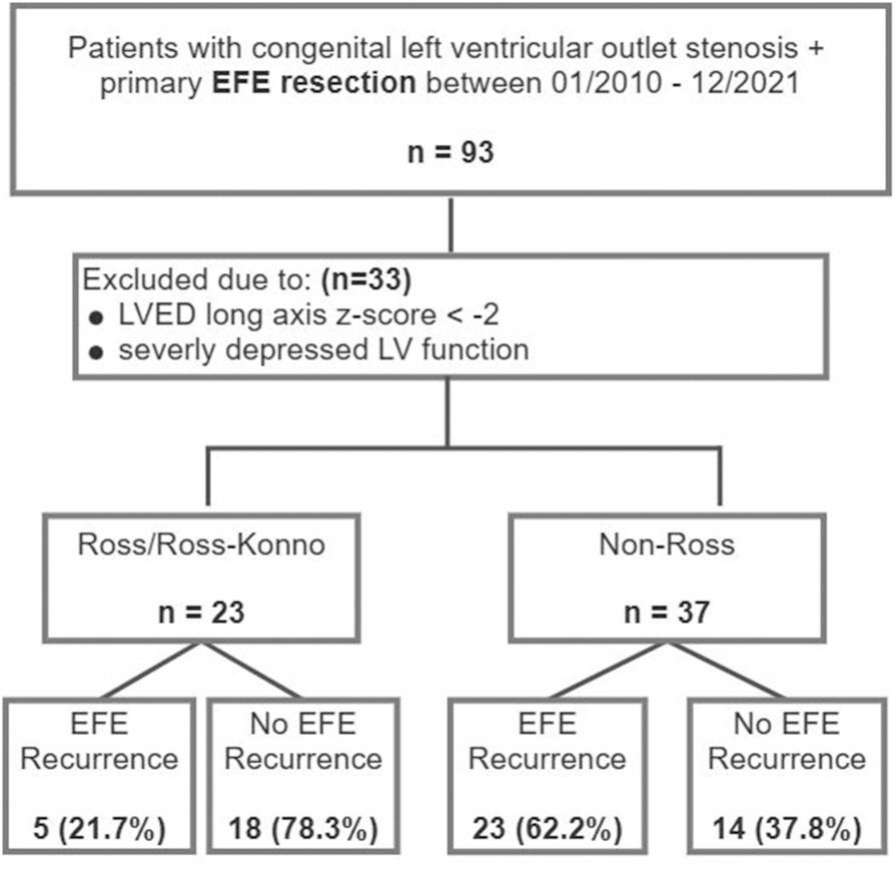
The primary outcome (i.e. EFE recurrence) was met by patients of the Study Group. Patients with hypoplastic left ventricles and those with severely depressed LV function were excluded. EFE, endocardial fibroelastosis; LVED, left ventricular end diastolic; MV, mitral valve; MS, mitral stenosis; SV, single ventricle; LV, left ventricle; BiV, biventricular.

During the study period, 93 patients diagnosed with AoV stenosis ± LVOT obstruction at birth underwent primary LV EFE resection at one of the two study centres. Of these, 60/93 patients (64.5%) met all inclusion criteria based on preoperative echocardiographic measurements (Fig. 1). In addition, 23/60 (38.3%) underwent a Ross/Ross**-**Konno procedure at or before primary EFE resection, while 37/60 (61.7%) did not receive a Ross procedure at that time (i.e. non-Ross group). Demographics and patient characteristics are detailed in Table 1. Concomitant procedures at primary EFE resection are listed in Table 2.

**Table 1:** Demographics and clinical characteristics of the study groups

	Ross/Ross-Konno (n = 23)	Non-Ross (n = 37)	SMD	*P* value
Age at primary EFE resection	0.23y (IQR: 0.04, 0.89)	2.26y (IQR: 0.89, 7.87)	0.83	<0.001
Male	17 (73.9)	33 (89.2)	0.4	0.161
Female	6 (26.1)	4 (10.8)		
Diagnosis			0.44	0.163
Critical AS	12 (52.2)	27 (73.0)		
Shone complex	11 (47.8)	10 (27.0)		
Fetal BVP	12 (52.2)	17 (45.9)	0.12	0.791
AoV annulus (z-score)	−1.68 (IQR: −2.77, −1.13)	−1.44 (IQR: −2.11, −0.31)	0.32	0.191
Aortic regurgitation ≥ mild	14 (60.9)	23 (62.2)	0.03	0.999
Neonatal aortic ballonvalvuloplasty	18 (78.3)	25 (67.6)	0.24	0.557
LVED long axis z-score	0.54 (IQR: −1.26, 1.94)	0.52 (IQR: −0.76, 1.53)	0.05	0.563
MV area z-score	−0.41 (IQR: −0.97, 0.7)	0.35 (IQR: −0.65, 1.42)	0.22	0.116
Ventricular function-preoperative			0.08	0.999
Normal	19 (82.6)	31 (83.8)		
Mild dysfunction	1 (4.3)	2 (5.4)		
Moderate dysfunction	3 (13.0)	4 (10.8)		
EFE Grade at surgery			0.27	0.672
1	2 (8.7)	6 (16.2)		
2	10 (43.5)	17 (45.9)		
3	11 (47.8)	14 (37.8)		
Mitral valve disease	16 (69.6)	21 (56.8)	0.27	0.416
Residual ≥ moderate LV dysfunction (after primary resection)	3 (13.0)	4 (10.8)	0.07	0.999

Values are presented as median (interquartile range) or frequency (%). AoV, aortic valve; AS, aortic stenosis; BVP, balloon valvuloplasty; EFE, endocardial fibroelastosis; IQR, interquartile range; LV, left ventricle; MV, mitral valve; SMD, standardized mean difference. Absolute SMD values ≥ 0.2 are considered as indicating substantial imbalance between the two groups. *P* values were calculated using the Wilcoxon rank-sum test or Fisher’s exact test.

**Table 2:** Concomitant procedures at primary EFE resection

Procedure	Ross/Ross-Konno	Non-Ross
	Group (n = 23)	Group (n = 37)
Ross-Konno	15	
Ross	5	
Aortic valvuloplasty		33
Subaortic resection		13
Mitral valvuloplasty	11	19
Aortic arch repair	2	1
Biventricular conversion	1	3
Norwood stage I		2
Glenn		2
Super-Glenn^a^		3
Tricuspid valvuloplasty		2
RVPAC replacement	1	
Aortic valve replacement		1
(Ross/Ross-Konno before primary EFE resection)	3	

EFE, endocardial fibroelastosis; RVPAC, right ventricle to pulmonary artery conduit.

a Super-Glenn: Glenn physiology with additional shunt to left pulmonary artery to increase pulmonary blood flow [30].

**Figure 2:**
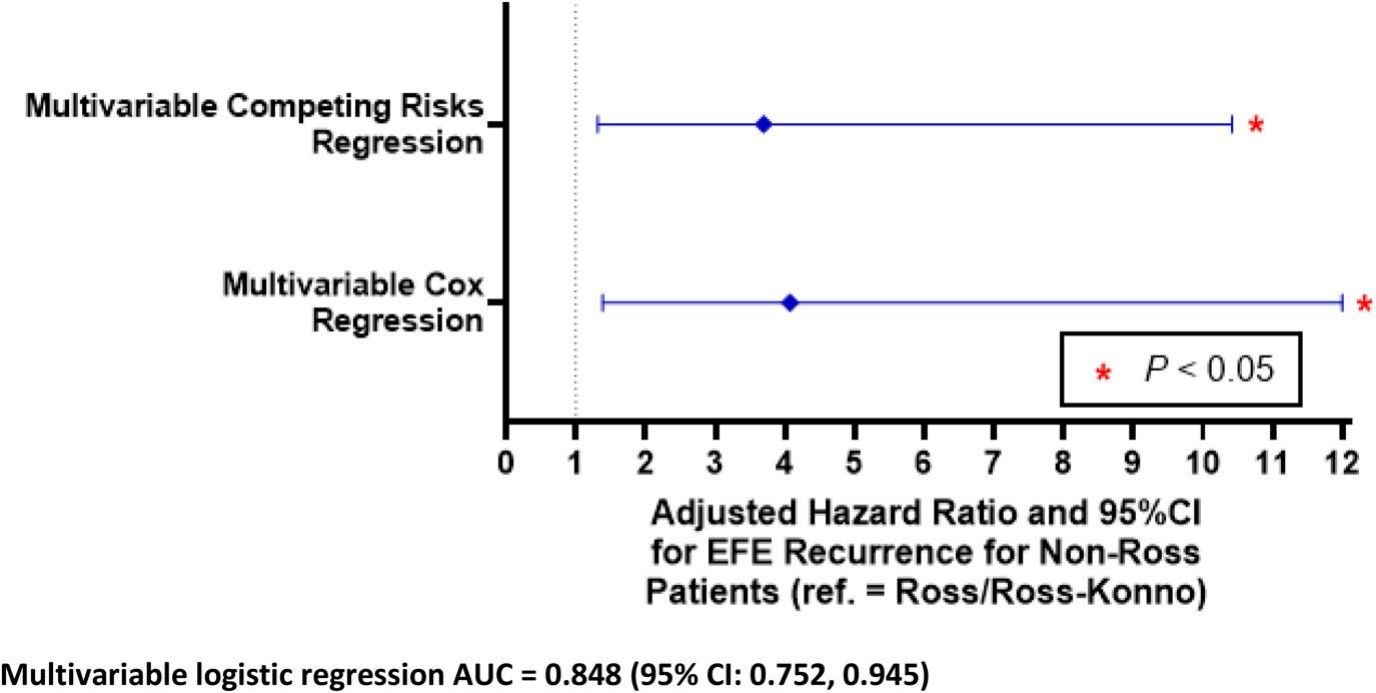
Multivariable competing risks and Cox regression models were used to predict primary outcome EFE recurrence in the Study Group: 5 out of 23 Ross/Ross**-**Konno patients and 23 out of 37 non-Ross patients developed EFE recurrence (i.e. primary outcome). CI, confidence interval; EFE, endocardial fibroelastosis.

**Figure 3:**
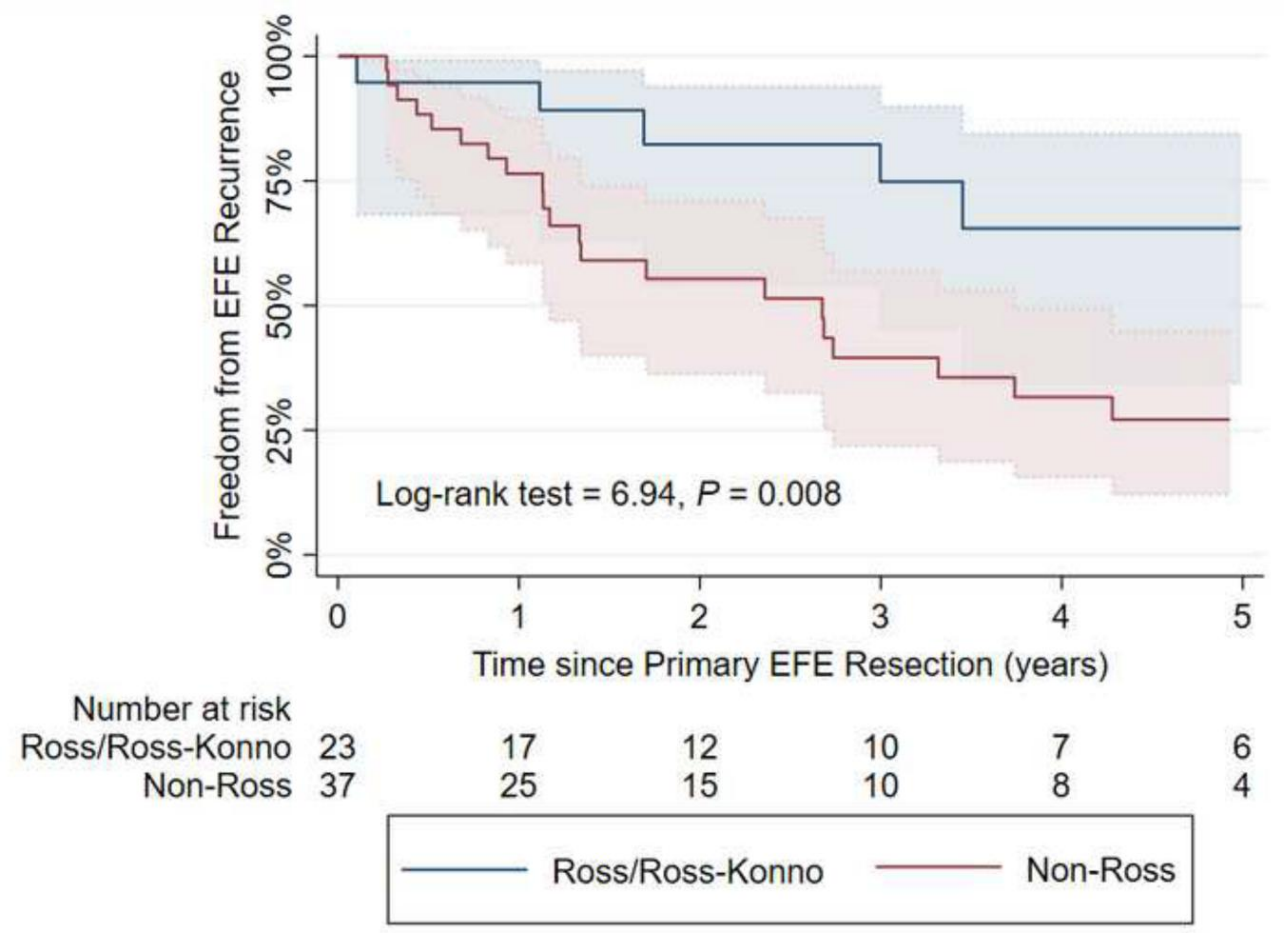
Estimated Kaplan–Meier freedom from EFE recurrence comparing Ross/Ross**-**Konno with non-Ross patients with 95% confidence intervals. EFE, endocardial fibroelastosis.

**Figure 4:**
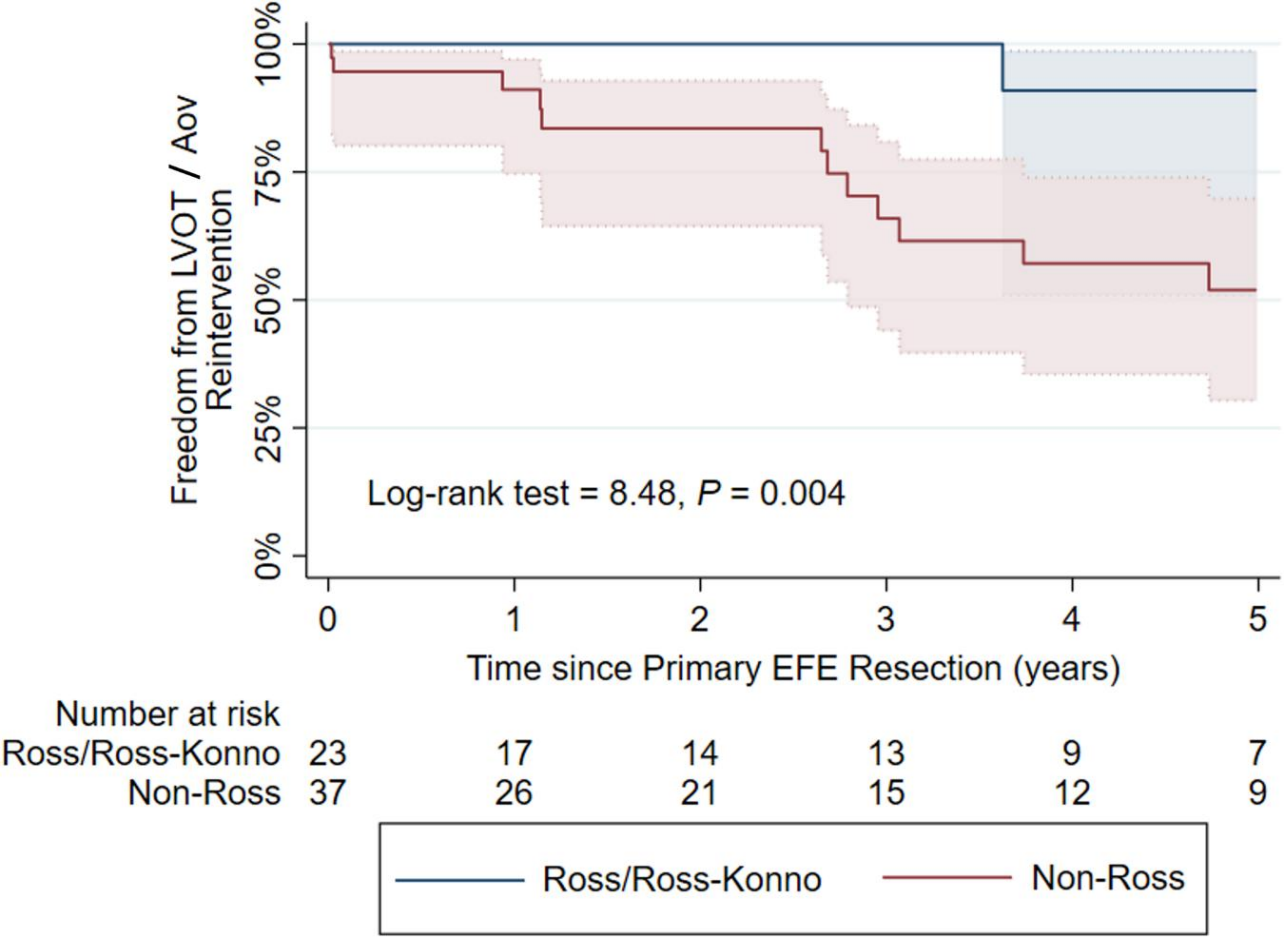
Estimated Kaplan–Meier freedom from reintervention on the left ventricular outflow tract/aortic valve with 95% confidence intervals. LVOT, left ventricular outflow tract; AoV, aortic valve.

**Figure 5:**
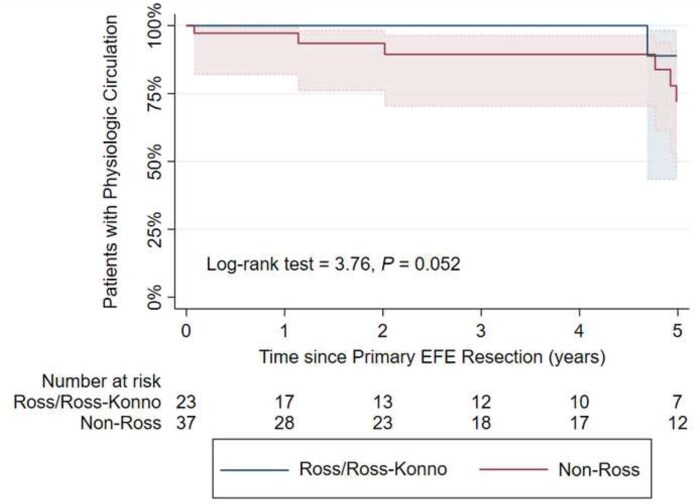
Estimated Kaplan–Meier patients with physiologic biventricular circulation with 95% confidence intervals. EFE, endocardial fibroelastosis.

In the Ross/Ross-Konno group, 5 out of 23 patients (21.7%) experienced EFE recurrence, compared to 23 out of 37 (62.2%) in the non-Ross group (difference = 40.5%, 95% CI 14.6–58.7, *P *= 0.003). Analysis of SMDs revealed imbalances (SMD ≥ 0.2) between the Ross/Ross-Konno group and non-Ross group of several variables (see Table 1), necessitating multivariable adjustment. The likelihood of EFE recurrence was higher in the non-Ross group, with an adjusted hazard ratio of 4.07 (95% CI 1.38–12.0, *P *= 0.011) and 3.69 (95% CI 1.31–10.42, *P *= 0.014) when accounting for death as a competing event (Fig. 2). This is equivalent to a risk reduction of 75% (adjusted HR: 0.25; 95% CI 0.08–0.72; *P *= 0.011) or 73% when accounting for death as a competing event (adjusted HR: 0.27; 95% CI 0.09–0.77; *P *= 0.014) in EFE recurrence for the Ross/Ross**-**Konno group. The median time to recurrence in the non-Ross group was 2.67 years (Fig. 3). Additionally, freedom from reintervention on the LVOT/AoV was significantly higher in the Ross/Ross**-**Konno group compared to non-Ross group (1/23, 4.4% vs 15/37, 40.5%; difference = 36.1%; 95% CI 15.4–53.4; log-rank test = 8.48, *P *= 0.004, Fig. 4). Of the patients in the Ross/Ross-Konno group, 78.3% (18/23) were alive with a physiologic biventricular circulation at the latest follow-up (Supplementary Material, Fig. S2); two patients died and two patients received heart transplantation due to ongoing severe mitral valve disease, and one required a reversed double switch operation due to severe LV dysfunction, for all patients at a median of 4.0 years after the primary EFE resection. In the non-Ross group, 26/37 (70.3%) patients had physiologic, biventricular circulation at the last follow-up. The remaining patients experienced five deaths, one ventricular assist device implantation, four patients in definite single ventricle physiology and one conversion to a reversed 1 1/2 ventricle repair (right ventricle supporting the systemic circulation) at a median time of 1.1 years after primary EFE resection. 72.5% (8/11) of patients with adverse outcomes in the non-Ross group had EFE recurrence. A trend was observed for patients undergoing the Ross/Ross**-**Konno procedure to achieve physiologic circulation more frequently (log-rank test = 3.76, *P *= 0.052, Fig. 5). The median total follow-up time of our cohort was 4.02 years (IQR: 0.99, 7.33).

### Evaluation of echocardiographic EFE assessment and independent echocardiographic evaluation of EFE recurrence

A detailed description of our independent and blinded analysis is outlined in the Supplemental Materials.

## DISCUSSION

Patients with congenital critical AS, potentially complicated by LVOTO and concomitant LV EFE, are at increased risk for adverse outcomes such as diastolic heart failure, definite single ventricle palliation, heart transplantation or death [17, 20, 31]. Studies of early biventricular repair and our recent investigation on LV recruitment and biventricular conversion identified these patients as high risk for adverse outcomes [9, 31]. We recently identified long-standing LV pressure overload as a risk factor for EFE recurrence, prompting this retrospective study focusing on the Ross/Ross**-**Konno procedure, as this is a particularly effective treatment strategy for addressing left ventricular outlet stenosis and significantly minimizing LV afterload [24].

The main findings of our study are that the relief of left ventricular outlet stenosis via the Ross/Ross-Konno procedure at the time of primary EFE resection substantially reduced the risk for EFE recurrence—up to 75%. Importantly, patients also benefited from a marked reduction in the need for reintervention on the LVOT/AoV. These findings validate our previous findings that unrelieved left ventricular outlet stenosis significantly contributes to EFE recurrence, resulting in LV diastolic dysfunction as EFE takes on a more infiltrative growth pattern [23].

Analysis of the SMD revealed that patients undergoing the Ross/Ross-Konno procedure had earlier primary EFE resection (at a median of 0.23 years), which might be due to better LV exposure after resection of the aortic root compared to EFE resection through the mitral valve or AoV in the non-Ross group. Although this was included in the multivariable adjustment to allow robust statistical analysis, the optimal timing of EFE resection has to be determined in future studies. The imbalance of other variables showed a higher rate of mitral valve disease, EFE burden and smaller AoV annuli in the Ross/Ross-Konno group, which assumes a more severe form of the disease and might be unfavourable regarding the primary outcome.

Critical AS, often associated with hypoplasia of left-sided structures, has traditionally eluded curative biventricular repair efforts. With a better understanding of the role of EFE in this disease process, LV rehabilitation has altered the trajectory of this congenital disease. Although EFE resection facilitates LV size adaptation, it rarely offers a permanent solution, as EFE frequently regrows. While a genetic predisposition may play a role, further studies are required to elucidate its impact definitively [24]. However, this study did not aim to determine the general eligibility and timing of a biventricular conversion in this patient cohort. All patients in this cohort had some degree of EFE, and therefore, they represent a selected group of patients, which might not be comparable with borderline left heart patients without EFE as ventricular function and growth might differ substantially (e.g. in patients with a concomitant VSD). Furthermore, major surgical interventions such as the Ross/Ross-Konno procedure have their own inherent risks of morbidity and mortality, especially when performed at a very young age, which cannot be ignored and should be accounted for in the individual decision-making process for each patient [32]. It remains to be determined whether diastolic function can be preserved in this patient cohort, which would have long-lasting benefits. As previously shown [24], decreased left ventricular filling due to small left ventricular size and decreased function (and thus relative flow stagnation) of the LV might have its own risk of EFE recurrence. Therefore, we excluded hypoplastic LVs and those with severely decreased left ventricular function. Furthermore, those patients are commonly not offered a Ross-Konno procedure/biventricular physiology pathway due to an inadequately sized LV.

Our findings underscore the necessity of incorporating strategies to target EFE when pursuing corrective biventricular repair in this patient cohort. While surgical interventions remain central, adjunctive therapies are needed to prevent EFE recurrence. Since we have already established the underlying cellular mechanism of EFE development, we explored potential pharmacological therapies, such as losartan and atorvastatin, which have shown promising results in preclinical studies [33, 34]. Prospective trials are essential to refine the patient selection, determine the optimal timing of a Ross/Ross-Konno procedure and mitigate other risk factors identified for EFE recurrence.

## CONCLUSION

This study found that patients after a Ross/Ross-Konno procedure were less likely to experience EFE recurrence and reinterventions on the LVOT/AoV were significantly reduced. However, patient selection and timing of a Ross/Ross-Konno procedure to prevent EFE recurrence have yet to be identified through prospective trials.

### Limitations of the study

An inherent problem is associated with retrospective analyses, which limits echocardiographic data for assessing EFE recurrence to those available at any given time. Because of the lack of standardized EFE diagnosis by echocardiography, two independent observers reassessed the presence or absence of EFE to mitigate this limitation best. Another concern is statistical power when investigating rare congenital cardiac defects and the heterogeneity of our patient cohort. Our remedy was to combine the experience of two centres treating higher volumes of these patients. There is potential bias due to better exposure for EFE resection in patients undergoing aortic root replacement surgery and minor inter-centre variations concerning some technical aspects of the procedures cannot be excluded due to the retrospective nature of the study.

## Supplementary Material

ezaf214_Supplementary_Data

## Data Availability

The data underlying this article will be shared on reasonable request to the corresponding author.

## ABBREVIATIONS

AoV: Aortic valve
AS: Aortic stenosis
CI: Confidence interval
CMR: Cardiac magnetic resonance
EFE: Endocardial fibroelastosis
HLHS: Hypoplastic left heart syndrome
IQR: Interquartile range
LGE: Late gadolinium enhancement
LV: Left ventricle
LVOT: Left ventricular outflow tract
LVOTO: Left ventricular outflow tract obstruction
SMD: Standardized mean difference
