# Supplementary material for: Eliminating left ventricular outlet stenosis lowers the risk for endocardial fibroelastosis recurrence
Source: Eur J Cardiothorac Surg. 2025 Jun 26;67(7):ezaf214. doi: 10.1093/ejcts/ezaf214 (PMC12254124; doi:10.1093/ejcts/ezaf214)
Supplement: ezaf214_Supplementary_Data [file ezaf214_supplementary_data.zip › Supplements.docx]

**Supplemental materials of: Eliminating left ventricular outlet stenosis lowers the risk for endocardial fibroelastosis recurrence**

Gregor Gierlinger^1,2,3^, Daniel Diaz-Gil^1,4,5,6,7^, Andreas Tulzer^3,8^, Roland Mair^2^, Eva Sames-Dolzer^2,3^, Kerstin Saraci^1^, Steven J. Staffa^9^, David Zurakowski^9,10^, Michaela Kreuzer^2,3^, Fabian Seeber^2^, Sitaram M. Emani^1,11^, Pedro J. del Nido^1,11^, Rudolf Mair^2^, Ingeborg Friehs^1,11^

^1^ Department of Cardiac Surgery, Boston Children’s Hospital, Boston, MA, USA

^2^ Division of Pediatric and Congenital Heart Surgery, Kepler University Hospital, Linz, Austria

^3^ Medical Faculty, Johannes Kepler University Linz, Altenberger Strasse 69, 4040 Linz, Austria

^4^ Department of Pediatrics, Boston Children’s Hospital, Boston, MA, USA

^5^ Department of Pediatrics, Harvard Medical School, Boston, MA, USA

^6^ Department of Pediatrics, Boston University Chobanian & Avedisian School of Medicine, Boston, MA, USA

^7^ Department of Pediatric Heart Medicine and Adults with Congenital Heart Disease, University Heart and Vascular Center, University Medical Center Hamburg-Eppendorf, Hamburg, Germany

^8^ Children's Heart Center Linz, Department of Pediatric Cardiology, Kepler University Hospital, Linz, Austria.

^9^ Department of Anesthesiology, Critical Care, and Pain Medicine, Boston Children's Hospital, Boston, MA, USA

^10^ Department of Anesthesia, Harvard Medical School, Boston, MA, USA

^11^ Department of Surgery, Harvard Medical School, Boston, MA

**EFE Diagnosis and Grading**

Preoperative CMR with late gadolinium enhancement as the gold standard for EFE diagnosis was available only in a subset of these patients (54/93) and was reviewed to assist with grading of EFE extent. However, preoperative echocardiographic images were available for retrospective review and EFE graded for all patients as described below. The definite diagnosis of LV EFE and inclusion in the study was derived from surgery reports and intraoperative description of the primary EFE resection procedure. The grading at the time of primary resection was determined by combining unblinded CMR reports, echocardiography, and a review of the surgical reports. A score based on the criteria published by McElhinney et al. (1) was used to grade EFE with: 0 = no EFE in the LV (this includes patients who had EFE on the atrial surface adjacent to the mitral valve or left atrium only); 1 = EFE involvement of papillary muscles only or selectively in the LVOT; 2 = noncontinuous patches throughout the LV ± papillary muscles; 3 = severe, continuous EFE lining throughout the LV.

**Echocardiographic EFE assessment**

To verify the validity of echocardiographic EFE assessment, preoperative echocardiographic images were reassessed and EFE graded by one blinded observer. The score (0-3) was compared to the unblinded evaluation of preoperative EFE grading, and an inter-observer agreement was measured using Cohen’s kappa coefficient to compare the interrater reliability (2).

**Independent Evaluation of EFE Recurrence**
Following primary EFE resection, postoperative (pre-discharge) echocardiographic images

were used as reference to assess EFE recurrence at follow-up or before re-resection, as applicable (Supplementary Figure 1). Special attention was given to residual EFE within the LV. A blinded evaluation of EFE grading from the most recent follow-up (or before re-resection, as applicable) was performed. Cohen’s kappa was again used to compare interrater reliability.

In addition to grading, the LV was evaluated for recurrence of EFE between the postoperative (pre-discharge) echocardiographic images and the follow-up (or before re-resection) images. Additional EFE areas in the LV, increasing thickness, and recurrence at primary resected areas were studied by one unblinded and one blinded observer. Cohen’s kappa was calculated for this binary measure (recurrence/no recurrence). In cases where the evaluations between these two observers did not match, a second blinded observer was asked to evaluate the echocardiographic images for final decision.

**Evaluation of Echocardiographic EFE Assessment**

Comparative analysis of preoperative blinded EFE grading (0-3) from echocardiographic images to the unblinded evaluation showed a weighted kappa of 0.64 (*P* < 0.001) with an observed weighted agreement of 89.1%. The blinded observer overrated the extent of EFE in six cases, rating grade 3, although EFE was less severe in the unblinded review of CMR, echocardiography, and surgical notes.

**Independent Echocardiographic Evaluation of EFE Recurrence**

Further comparison of EFE grading at the last available echocardiographic follow-up (or just before re-resection if applicable) between unblinded and blinded observers showed a weighted kappa coefficient of 0.74 (*P* < 0.001) and an observed weighted agreement of 93.3%.

EFE recurrence as the primary outcome measure was evaluated between postoperative (pre-discharge) echocardiographic images and follow-up (or pre re-resection, as applicable) images by the unblinded and blinded observer, and the comparison showed a kappa value of 0.70 *(P* < 0.001) and an observed weighted agreement of 85.0%. In a total of 9/60 cases, the unblinded and blinded evaluation of EFE recurrence did not match, as the blinded evaluation led to the diagnosis of EFE recurrence in 6 more cases than the unblinded evaluation. These nine follow-up echocardiographic images were reviewed by a second blinded observer who agreed to 3/9 EFE recurrences as the final decision.

**Supplemental Figure Legends:**

**Supplemental Figure 1:**

Timeline of the independent evaluation of EFE recurrence. EFE, endocardial fibroelastosis; CMR, cardiac magnetic resonance.

**Supplemental Figure 2**:

Secondary outcome: failure of the left ventricle to be considered a systemic ventricle. HTX, heart transplantation; LVAD, left ventricular assist device; SV, single ventricle; BiV, biventricular.

**Supplemental References**

1. McElhinney DB, Vogel M, Benson CB et al. Assessment of left ventricular endocardial fibroelastosis in fetuses with aortic stenosis and evolving hypoplastic left heart syndrome. *Am J Cardiol*. 2010; 106(12):1792-1797
2. Cohen J. A coefficient of agreement for nominal scales. *Educational and Psychological Measurement.* 1960; Vol. XX, No. 1
