# Supplementary figures and images for: Eliminating left ventricular outlet stenosis lowers the risk for endocardial fibroelastosis recurrence
Source: Eur J Cardiothorac Surg. 2025 Jun 26;67(7):ezaf214. doi: 10.1093/ejcts/ezaf214 (PMC12254124; doi:10.1093/ejcts/ezaf214)

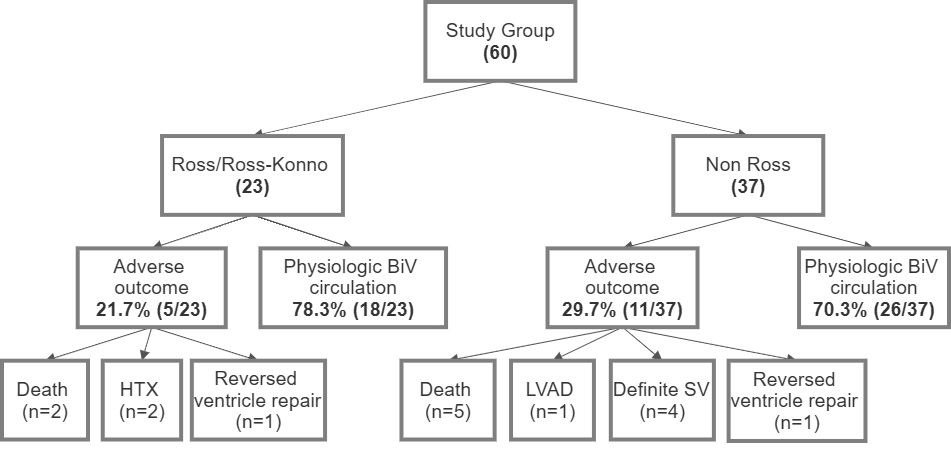

Supplement: ezaf214_Supplementary_Data [file ezaf214_supplementary_data.zip › Supp Fig 2.jpg]

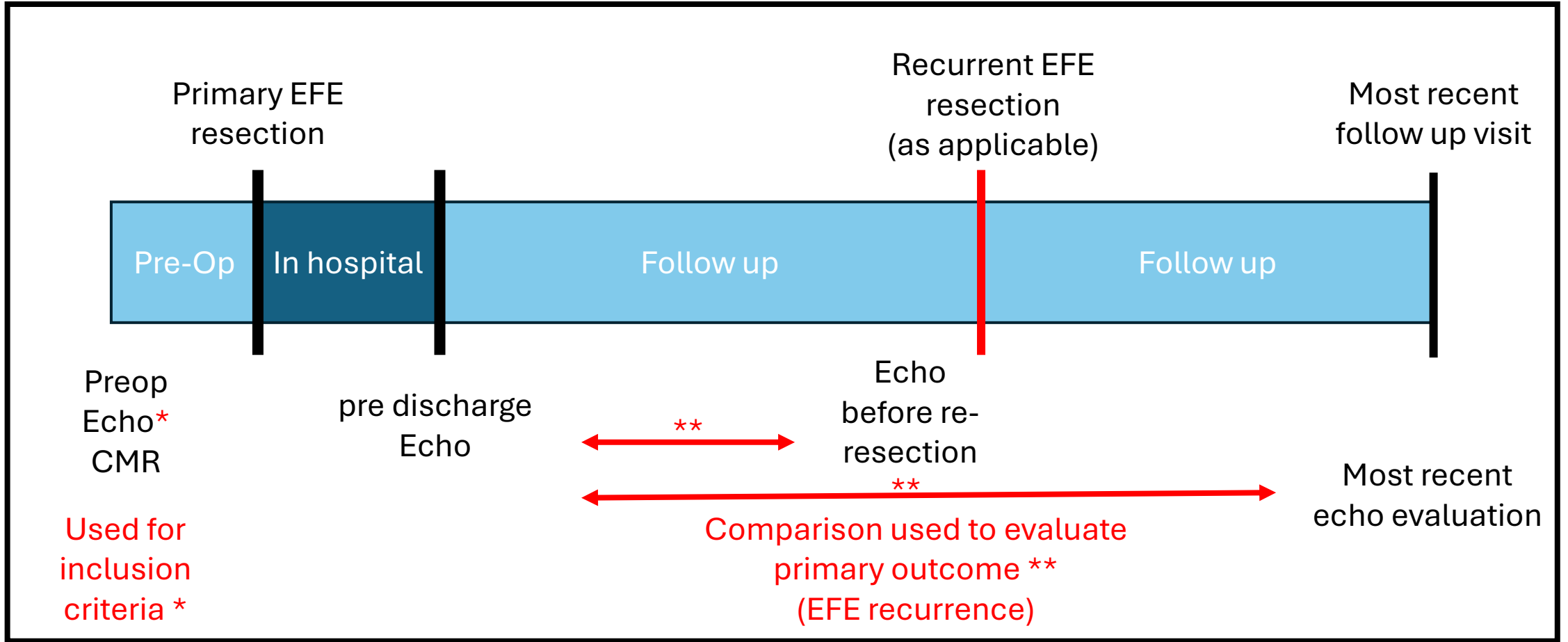

Supplement: ezaf214_Supplementary_Data [file ezaf214_supplementary_data.zip › Supplementary Figure 1.pdf]
